# Porcine Deltacoronavirus Infection Disrupts the Intestinal Mucosal Barrier and Inhibits Intestinal Stem Cell Differentiation to Goblet Cells via the Notch Signaling Pathway

**DOI:** 10.1128/jvi.00689-23

**Published:** 2023-06-08

**Authors:** Shuai Zhang, Shuoshuo Zhang, Yuchen Hou, Yanjie Huang, Jiajia Cai, Guangzheng Wang, Yanan Cao, Zhenhai Chen, Xiaomin Fang, Wenbin Bao

**Affiliations:** a College of Animal Science and Technology, Yangzhou University, Yangzhou, China; b College of Veterinary Medicine, Yangzhou University, Yangzhou, China; c Joint International Research Laboratory of Agriculture & Agri-Product Safety of MOE, Yangzhou University, Yangzhou, China; d Institute of Food Safety and Nutrition, Jiangsu Academy of Agricultural Sciences, Nanjing, China; Instituto de Biotecnologia/UNAM

**Keywords:** Notch signaling pathway, PDCoV, goblet cells, intestinal organoids, intestinal stem cells

## Abstract

Goblet cells and their secreted mucus are important elements of the intestinal mucosal barrier, which allows host cells to resist invasion by intestinal pathogens. Porcine deltacoronavirus (PDCoV) is an emerging swine enteric virus that causes severe diarrhea in pigs and causes large economic losses to pork producers worldwide. To date, the molecular mechanisms by which PDCoV regulates the function and differentiation of goblet cells and disrupts the intestinal mucosal barrier remain to be determined. Here, we report that in newborn piglets, PDCoV infection disrupts the intestinal barrier: specifically, there is intestinal villus atrophy, crypt depth increases, and tight junctions are disrupted. There is also a significant reduction in the number of goblet cells and the expression of *MUC-2*. *In vitro*, using intestinal monolayer organoids, we found that PDCoV infection activates the Notch signaling pathway, resulting in upregulated expression of *HES-1* and downregulated expression of *ATOH-1* and thereby inhibiting the differentiation of intestinal stem cells into goblet cells. Our study shows that PDCoV infection activates the Notch signaling pathway to inhibit the differentiation of goblet cells and their mucus secretion, resulting in disruption of the intestinal mucosal barrier.

**IMPORTANCE** The intestinal mucosal barrier, mainly secreted by the intestinal goblet cells, is a crucial first line of defense against pathogenic microorganisms. PDCoV regulates the function and differentiation of goblet cells, thereby disrupting the mucosal barrier; however, the mechanism by which PDCoV disrupts the barrier is not known. Here, we report that *in vivo*, PDCoV infection decreases villus length, increases crypt depth, and disrupts tight junctions. Moreover, PDCoV activates the Notch signaling pathway, inhibiting goblet cell differentiation and mucus secretion *in vivo* and *in vitro*. Thus, our results provide a novel insight into the mechanism underlying intestinal mucosal barrier dysfunction caused by coronavirus infection.

## INTRODUCTION

Porcine deltacoronavirus (PDCoV), a member of the genus *Deltacoronavirus*, primarily infects newborn piglets, causing severe diarrhea, vomiting, and dehydration (1). It is an emerging enteropathogenic coronavirus that has caused large economic losses to pork-producing industries worldwide (2). PDCoV is highly contagious and is transmitted mainly through the fecal-oral route, but it can be transmitted by aerosols (3); it is also considered a threat to global health due to its potential for cross-species transmission (4–6). PDCoV has a single-stranded positive-sense RNA genome that encodes four structural proteins, the spike (S), nucleocapsid (N), envelope (E), and membrane (M) proteins (7). Like other enteric coronaviruses, such as porcine epidemic diarrhea virus (PEDV) and transmissible gastroenteritis virus (TGEV), PDCoV mainly infects the small intestine of pigs, although newborn piglets are the most susceptible and the most seriously sickened, with a high mortality rate (2, 8).

The small intestine is one of the principal sites of food digestion and nutrient absorption in piglets, and the integrity of intestinal villi is an important factor in nutrient absorption (9). Normally, the intestinal epithelium is in a state of dynamic balance; epithelial cells differentiate directionally into various mature epithelial cells with absorptive function (enterocytes) or secretory function (goblet cells, Paneth cells, and endocrine cells) during an upward migration along the crypt-villus axis (10). A constant stream of regenerating cells replenishes or replaces apoptotic and shed cells, maintaining the integrity of the intestinal epithelium. Goblet cells, a specialized secretory type of epithelial cell, are a key component of the intestinal mucosal barrier (11), serving as the main cellular component of the innate defense system (12–14). In addition to synthesizing and secreting the mucin (MUC) family of proteins, which constitute the most important component of intestinal mucus (15), goblet cells also secrete a variety of antimicrobial proteins, including Fc gamma binding protein (16), anterior gradient 2 (17), and zymogen granule 16 (18). With this defense arsenal, intestinal mucus barriers are essential for holding microbes, viruses, and other luminal contents at a safe distance from the underlying epithelium. These mucus barriers also create a self-protective microenvironment against self-digestion (15). When goblet cells in the intestine are deficient or dysfunctional in mucus secretion, the intestinal mucus layer becomes thin and fails to provide effective protection against intestinal diseases. Growing evidence indicates that enteroviruses modulate the formation and function of goblet cells. For example, human adenoviruses preferentially infect goblet cells (19), astrovirus, a global cause of pediatric diarrhea, infects goblet cells and alters the intestinal mucus barrier (20), and enterovirus 71 preferentially infects goblet cells and reduces the expression of goblet cell-derived mucin (21). Even in the absence of direct infection, enteric viruses can indirectly influence the number and the function of goblet cells. Rotavirus infection causes apoptosis of intestinal epithelial cells (22). Goblet cell numbers decrease as a result of delaying intestinal repair. To date, the effects of PDCoV infection on goblet cells and their function remain unclear.

Intestinal stem cells are located at the bottom of the crypt and produce transit-amplifying (TA) progenitors. TA progenitors undergo terminal differentiation into distinct intestinal cells (enterocytes, goblet cells, Paneth cells, enteroendocrine cells, and tuft cells) under the regulation of signaling pathways (23). In the intestinal crypt, promotion of atonal basic helix-loop-helix (bHLH) transcription factor 1 (ATOH-1) and inhibition of Notch drive the development of secretory progenitor cells (24, 25). The Notch signaling pathway is a conserved intercellular communication signaling pathway that regulates goblet cell differentiation and is a key factor required for the proliferation and differentiation of intestinal stem cells during development (26, 27).

Herein, we report that in PDCoV-infected newborn piglets, the virus impairs the intestinal barrier and disrupts both proliferation and differentiation of intestinal stem cells. Specifically, PDCoV infection results in goblet cell loss and decreased secretion of mucus, leading to the destruction of the intestinal mucosal barrier. We also show that *in vivo* and *in vitro*, PDCoV infection activates the Notch signaling pathway and upregulates the expression of *Notch-1*, *JAG-1*, *Dll-4*, and *HES-1*, thereby suppressing the differentiation of intestinal stem cells to goblet cells. The results demonstrated that PDCoV infection impairs the intestinal mucosal barrier by regulating the differentiation of goblet cells and affecting the mucus secretion of goblet cells.

## RESULTS

### PDCoV infection disrupts the intestinal barrier.

**FIG 1:**
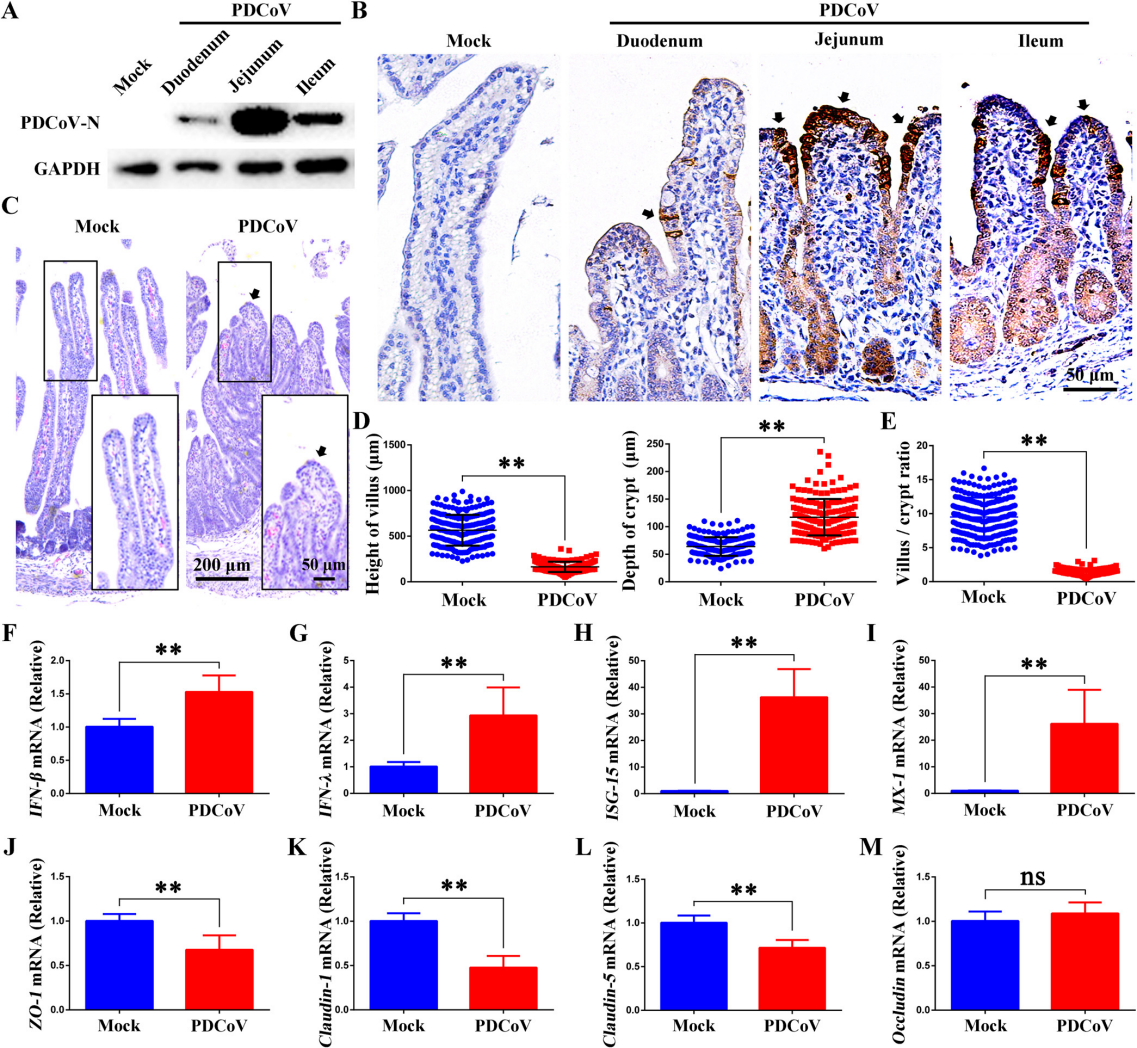
PDCoV infection impairs the intestinal barrier *in vivo*. (A) Western blot of PDCoV-N protein in small intestinal segments of infected and uninfected piglets. (B) Tissue tropism for PDCoV in duodenal, jejunum, and ileum segments determined by immunohistochemistry. PDCoV-N protein is stained deep yellow-brown (black arrows). Scale bar = 50 μm. (C) H&E staining of jejunum segments of uninfected and infected piglets. The black arrow indicates damaged villus. Scale bar = 200 μm or 50 μm. (D) Villus height and crypt depth. (E) The villus/crypt ratio. (F to I) *IFN-β*, *IFN-λ*, *ISG-15*, and *MX-1* mRNA levels in jejunum tissues. (J to M) *ZO-1*, *claudin-1*, *claudin-5*, and *occludin* mRNA levels in jejunum tissues. *, *P < *0.05; **, *P < *0.01. ns, not significant.

The data in Fig. 1 show the tissue tropism of PDCoV (CHN-GD16-05) in the small intestine of infected piglets. The Western blot (Fig. 1A) and immunohistochemical (IHC) stain (Fig. 1B) show that PDCoV primarily infects the epithelial cells of the jejunum. Hematoxylin and eosin (H&E) staining and histological analysis (Fig. 1C to E), performed to evaluate the effect of PDCoV infection on intestinal architecture, revealed that PDCoV infection results in a dramatic shortening of villus height and an increase in crypt depth, indicating that intestinal homeostasis is disrupted by PDCoV infection.

During viral infection, the host immune response is triggered to produce interferons (IFNs), thereby eliciting an antiviral state in infected cells and uninfected neighbor cells. Of note, intestinal epithelial cells are potent producers of type III interferon (28). Therefore, we quantitated by reverse transcription-quantitative real-time PCR (RT-qPCR) the mRNA levels of *IFN-β*, *IFN-λ*, and the downstream interferon-stimulated genes (ISGs) *ISG-15* and *MX-1* (Fig. 1F to I). We found that in the jejunum tissues of infected piglets, expression of these IFNs and ISGs was significantly increased over that in the tissues of uninfected piglets. Additionally, the mRNA levels of tight-junction markers *ZO-1*, *claudin-1*, *claudin-5*, and *occludin* were determined by RT-qPCR. These results showed that in the jejuna of infected piglets, the expression of *ZO-1*, *claudin-1*, and *claudin-5* was significantly decreased over that in the jejuna of uninfected piglets (Fig. 1J to L). There was no significant difference in the levels of *occludin* (Fig. 1M). For the purpose of maintaining intestinal homeostasis, the intestinal epithelium is a highly organized structure that is constantly being renewed from intestinal stem cells (ISCs) (29). Our results suggest that PDCoV infection impairs epithelial homeostasis and integrity by damaging the intestinal epithelial barrier and inhibiting intestinal stem cell proliferation.

### Loss of goblet cells in the intestinal villi of infected piglets.

**FIG 2:**
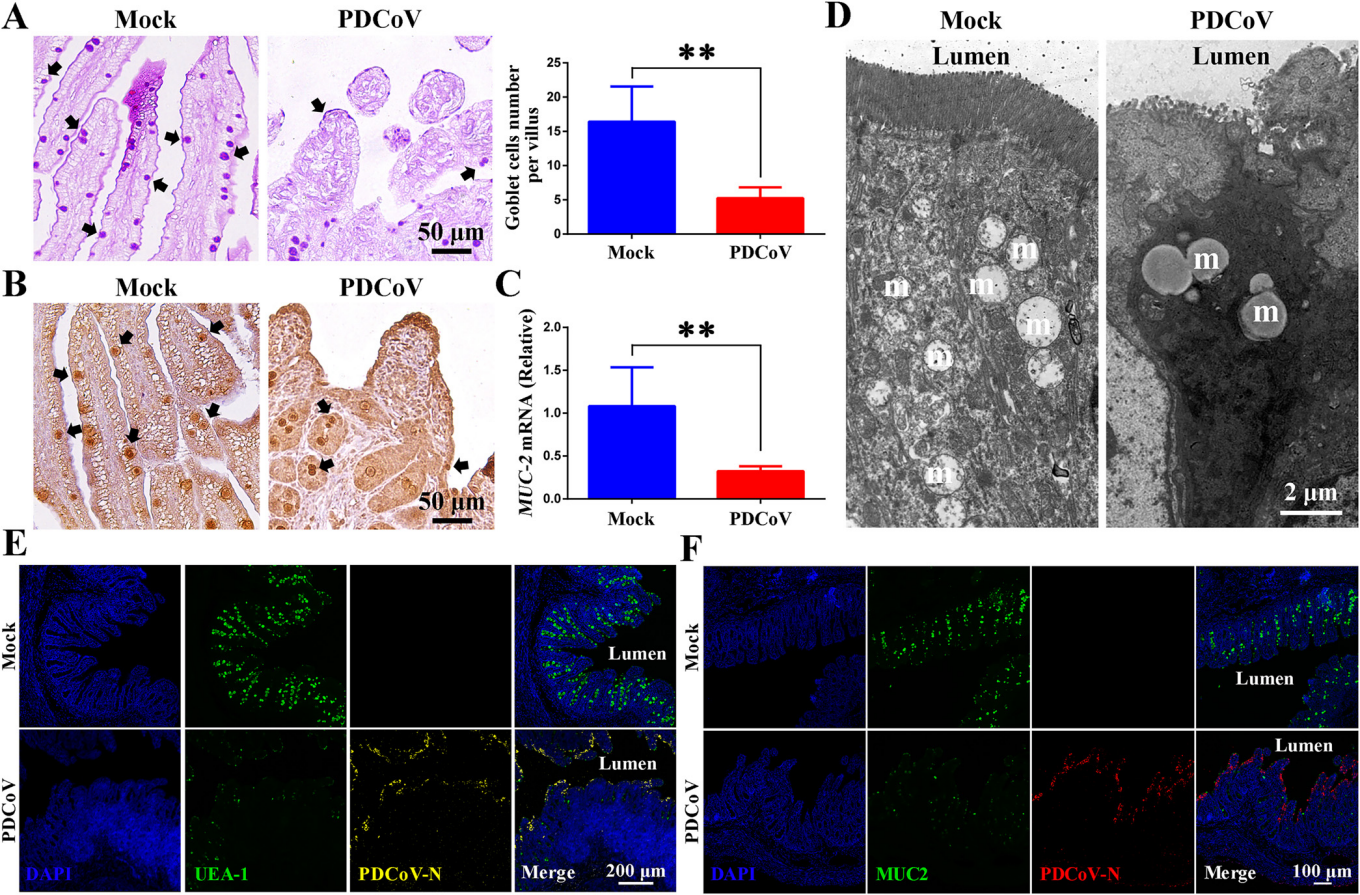
PDCoV infection of newborn piglets causes the loss of goblet cells from intestinal villi, thereby resulting in decreased mucus secretion. (A) Jejunum tissues were stained with periodic acid-Schiff (PAS) to quantitate goblet cells per villus (black arrows). Scale bar = 50 μm. (B) Jejunum tissues were stained for MUC-2 (black arrows). Scale bar = 50 μm. (C) *MUC-2* mRNA levels in homogenized jejunum tissues. (D) The mucus granules (m) in goblet cells were observed by transmission electron microscopy (TEM). Scale bar = 2 μm. (E) UEA-1^+^ cells (green) and PDCoV (yellow) in jejunum. Scale bar = 200 μm. (F) MUC-2^+^ cells (green) and PDCoV (red) in jejunum. Scale bar = 100 μm. **, *P < *0.01.

Goblet cells synthesize and secrete mucus, which plays an important role in inhibiting virus infection (14). Jejunum tissues stained for periodic acid-Schiff (PAS) analysis reveal that the population of goblet cells was significantly reduced in PDCoV-infected piglets (Fig. 2A). Consistent with this result, MUC-2 levels (a major component of mucus) were also significantly reduced in the infected piglets (Fig. 2B and C). Ultrastructural analysis of the goblet cells also showed a significant decrease in the number of mucus granules, and those present had an abnormal fused appearance (Fig. 2D). Confocal microscopy analysis of Ulex europaeus agglutinin 1 (UEA-1) and MUC-2 staining confirmed a marked reduction in intestinal mucus in the villi of PDCoV-infected piglets (Fig. 2E and F). These results demonstrate that PDCoV infection results in a reduced number of goblet cells and hence the secretion of mucus *in vivo*.

### PDCoV infection activates the Notch signaling pathway of porcine intestinal stem cells *in vivo*.

**FIG 3:**
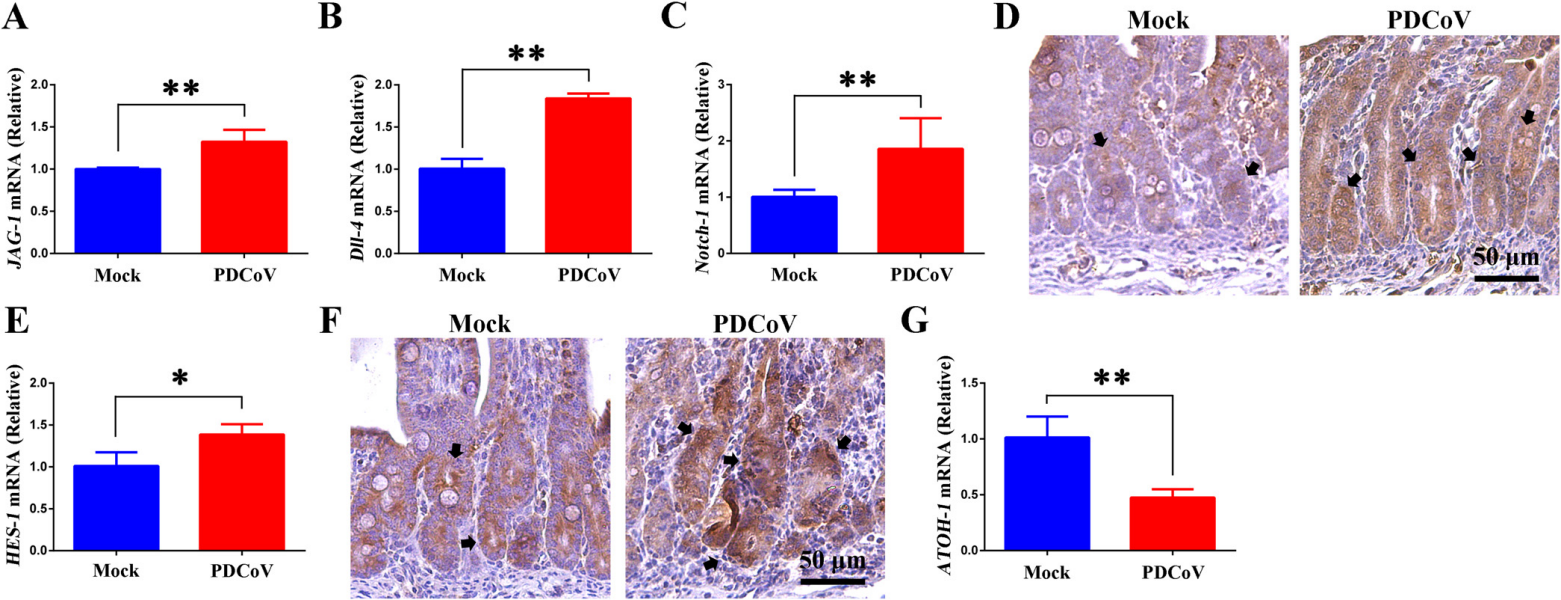
PDCoV infection activates the Notch signaling pathway in porcine intestinal stem cells *in vivo*. (A to C) *JAG-1* (A), *Dll-4* (B), and *Notch-1* (C) mRNA levels in homogenized jejunum tissues. (D) Notch-1 immunohistochemistry staining of jejunum sections; the black arrows indicate the Notch-1 protein. Scale bar = 50 μm. (E) *HES-1* mRNA levels in homogenized jejunum tissues. (F) HES-1 immunohistochemistry staining of jejunum sections; the black arrows indicate the HES-1 protein. Scale bar = 50 μm. (G) *ATOH-1* mRNA levels in homogenized jejunum tissues. *, *P < *0.05; **, *P < *0.01.

The Notch signaling pathway is a highly conserved signaling system that determines the fate of differentiation of ISCs (30). Particularly, Notch signaling regulates ISC differentiation to the secretory lineage of intestinal cells, including goblet cells (25). To determine whether the reduction of goblet cells and mucus caused by PDCoV infection is regulated via the Notch signaling pathway *in vivo*, we quantitated the mRNA levels of *JAG-1* and *Dll-4* (Notch ligands) and *Notch-1* (Notch receptor) by RT-qPCR. From the homogenized jejunum tissues of piglets, we found that those from infected piglets had significantly higher levels of expression of each of these genes than those from uninfected piglets (Fig. 3A to C). Immunohistochemistry staining showed that PDCoV infection resulted in increased levels of Notch-1 in the crypt area (Fig. 3D). Levels of *HES-1* and *ATOH-1* in homogenized jejunum tissues were evaluated by RT-qPCR; jejunum sections were stained for hairy and enhancer of split 1 (HES-1). PDCoV infection resulted in upregulated expression of *HES-1* (Fig. 3E and F) and downregulated expression of *ATOH-1* (Fig. 3G). These results indicate that *in vivo*, PDCoV activates the Notch signaling pathway, thereby suppressing the differentiation of porcine ISCs into goblet cells.

### Development of porcine 3D intestinal organoids and established 2D monolayer organoids are susceptible to PDCoV.

**FIG 4:**
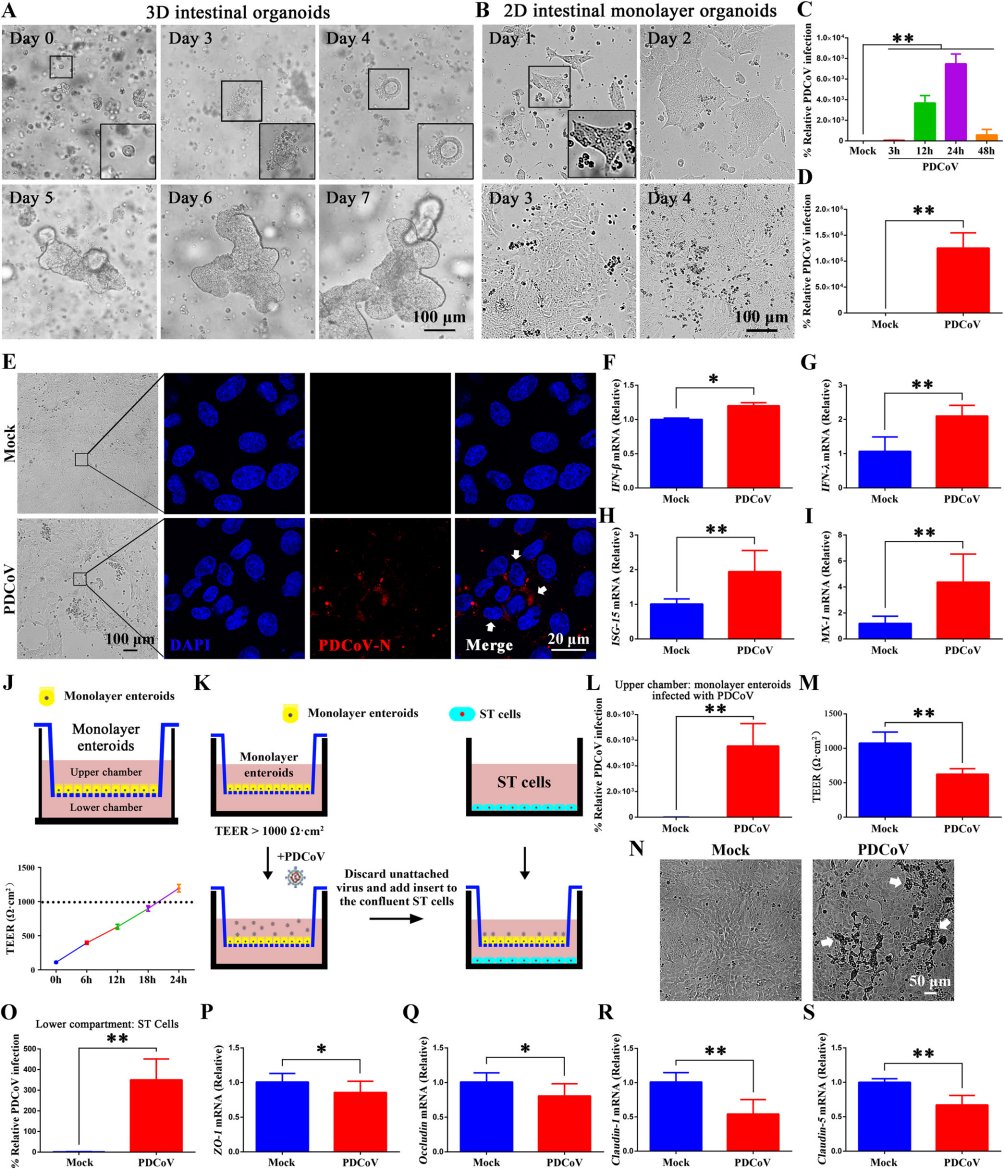
Porcine intestinal monolayer organoids are susceptible to PDCoV infection. (A) Development of porcine intestinal organoids from day 0 to day 7 as observed by light microscopy. (B) Growth of 2D intestinal monolayer organoids from day 1 to day 4 as observed by light microscopy. (C) Viral replication in 2D intestinal monolayer organoids was quantified by RT-qPCR at different times postinfection (MOI = 0.1). (D) Viral replication in intestinal monolayer organoids was quantified by RT-qPCR at 24 hpi (MOI = 1). (E) 2D intestinal monolayer organoids were infected with PDCoV (MOI = 1) for 24 h. PDCoV-N protein appears red and nuclei appear blue in IFA; cytopathic effect (CPE) could be seen by light microscopy. (F to I) The mRNA levels of *IFN-β* and *IFN-λ* and interferon-stimulated genes *ISG-15* and *MX-1* from uninfected and PDCoV-infected (MOI = 1) intestinal monolayer organoids. (J) Transepithelial electrical resistance (TEER) was measured across the monolayer enteroid. (K) Schematic of the Transwell coculture system for the monolayer enteroids and ST cells. (L) PDCoV-N mRNA levels from the upper chamber of monolayer enteroids were assessed by RT-qPCR. (M) TEER values from PDCoV and mock-infected monolayer enteroids. (N) CPE of PDCoV-infected ST cells (in the lower chamber) as observed by light microscopy. White arrows indicate the lesions in the ST cells. Scale bar = 50 μm. (O) PDCoV-N RNA levels of the ST cells from the lower chamber were assessed by RT-qPCR. (P to S) mRNA levels of tight-junction-related genes, *ZO-1*, *occludin*, *claudin-1*, and *claudin-5*, from uninfected and PDCoV-infected (MOI = 1) intestinal monolayer organoids. *, *P < *0.05; **, *P < *0.01.

To further investigate the influence of PDCoV infection on intestinal homeostasis and simulate the intestinal biology and host-pathogen interaction, porcine intestinal crypts were isolated from porcine jejunum and cultured to three-dimensional (3D) intestinal organoids following the procedure of Sato et al. (31). The cultured crypts gradually differentiated into budding spherical enteroids with a central lumen surrounded by an epithelium containing villus-like structures and budding crypt-like domains from small round cell clusters (Fig. 4A). Of note, the apical membrane of 3D enteroids faces internally; this makes it challenging to mimic the intestinal environment conducive to PDCoV infection. Thus, we also used a planar (two-dimensional) monolayer enteroid model to illustrate the kinetics of PDCoV infection (Fig. 4B). The porcine intestinal monolayer enteroids were collected at 3, 12, 24, and 48 h after PDCoV (multiplicity of infection [MOI] = 0.1) infection. The greatest relative level of PDCoV was observed 24 h postinfection (hpi) (Fig. 4C). This result may be due in part to the fact that many cells were dying or dead by 48 hpi. Figure 4D and E, respectively, show by RT-qPCR and immunofluorescence assay (IFA) that PDCoV (MOI = 1) can establish a productive infection on porcine intestinal monolayer organoids.

To assess the immune status of the intestinal monolayer organoids after PDCoV infection, the mRNA levels of type I and III interferons were quantified. Consistent with the results in piglets, the RT-qPCR results showed that in PDCoV-infected organoids, *IFN-β* and *IFN-λ* levels were elevated over those in mock-infected organoids (Fig. 4F and G). In addition, PDCoV infection also induced expression of interferon-stimulated genes *MX-1* and *ISG-15* (Fig. 4H and I). These results demonstrate that PDCoV elicits an immune response in intestinal monolayer organoids.

Transepithelial electrical resistance (TEER) is widely used as an indicator of the integrity of epithelial tissues and to assess the integrity and function of the intestinal epithelial barrier (32, 33). To further investigate the effect of PDCoV infection on the intestinal barrier, 3D enteroids were collected, mechanically dissociated, and then seeded in the upper chambers of Transwell culture plates. The plates were incubated under standard conditions and TEER was measured across the monolayer. TEER values reached >1,000 Ω·cm^2^ (indicating an intact monolayer with tight junctions) after 18 h (Fig. 4J). We next established a monolayer enteroid/ST cell coculture system (Fig. 4K). PDCoV infection caused lesions in the 2D monolayer enteroids (Fig. 4E and L) and disrupted the epithelial barrier, as indicated by the reduction of TEER in the infected 2D monolayer enteroids (Fig. 4M). The permeability of the 2D monolayer enteroid was assessed by monitoring the level of infected ST cells (located in the lower chamber). We found that after 24 h of incubation with PDCoV-infected enteroids, the ST cells showed obvious lesions (Fig. 4N) and were producing PDCoV N protein (Fig. 4O). The 2D monolayer enteroids also had lower levels of tight-junction-related genes (*ZO-1*, *occludin*, *claudin-1*, and *claudin-5*) (Fig. 4P to S). Together, these results demonstrate that the intestinal monolayer organoids we developed were susceptible to PDCoV and that their infection resulted in pathology similar to that observed *in vivo*.

### PDCoV infection results in fewer goblet cells and impairs mucus secretion via activating the Notch signaling pathway *in vitro*.

**FIG 5:**
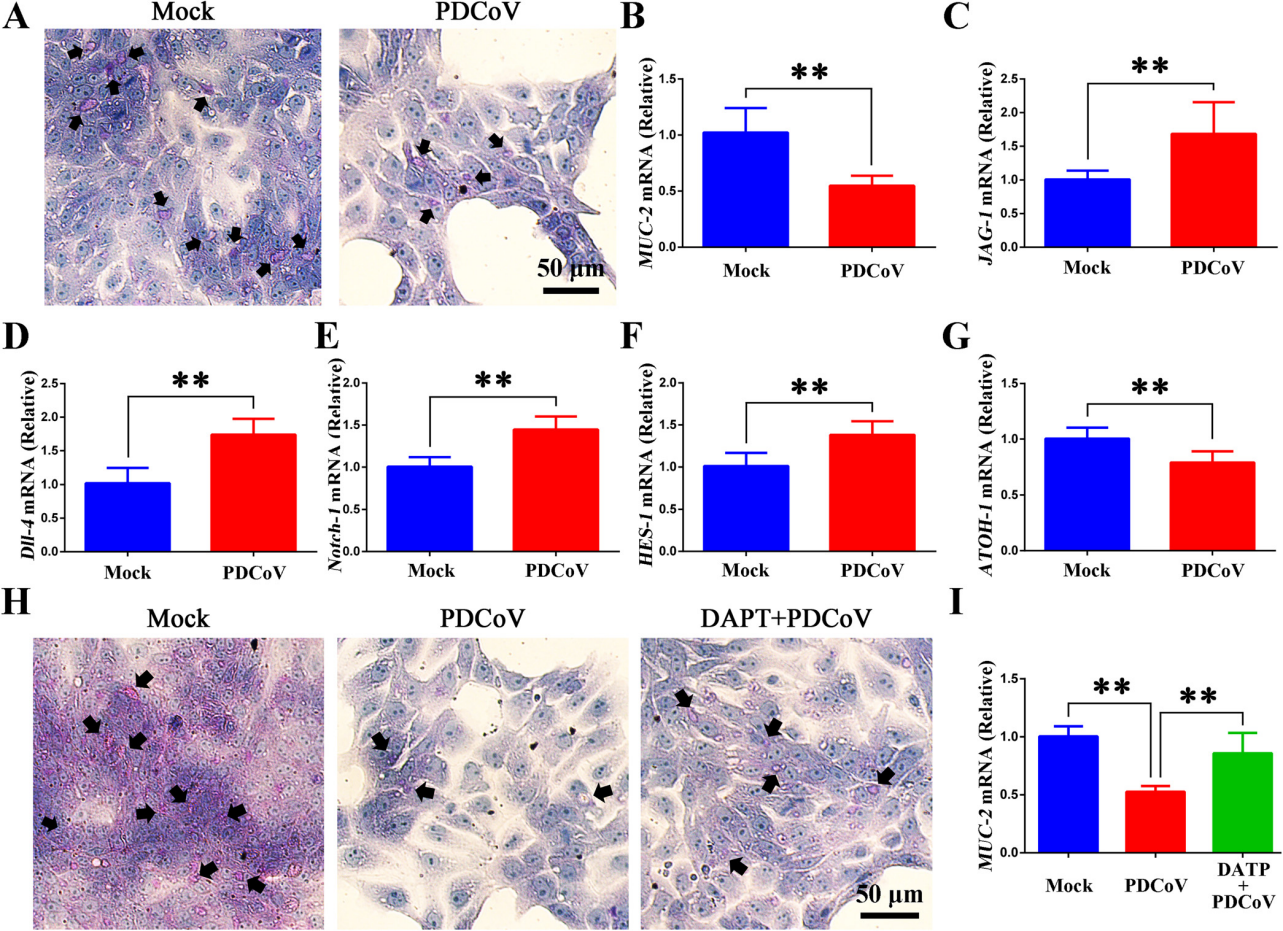
PDCoV infection activates the Notch signaling pathway in intestinal monolayer organoids. (A) Infected and uninfected intestinal monolayer organoids were stained with PAS; the black arrows indicate the goblet cells. (B) *MUC-2* mRNA levels from the intestinal monolayer organoids. (C to G) *JAG-1*, *Dll-4*, *Notch-1*, *HES-1*, and *ATOH-1* mRNA levels in uninfected and PDCoV-infected intestinal monolayer organoids. (H) Goblet cells (black arrows) of the intestinal monolayer organoid were stained with PAS. (I) *MUC-2* mRNA levels from the intestinal monolayer organoid. *, *P < *0.05; **, *P < *0.01.

ISCs differentiate into multiple types of cells, including enterocytes, goblet cells, enteroendocrine cells, and Paneth cells. As PDCoV infection in piglets resulted in a decreased population of goblet cells and hence decreased mucus production, we sought to determine if the same was true *in vitro*. Consistent with the results *in vivo*, cultured organoids infected with PDCoV (24 h; MOI = 1) had fewer goblet cells as assessed by PAS staining (Fig. 5A). Moreover, the level of *MUC-2* was also significantly decreased (Fig. 5B).

To determine if PDCoV infection activates the Notch signaling pathway in intestinal monolayer organoids, levels of *JAG-1*, *Dll-4*, *Notch-1*, and *HES-1* were quantitated by RT-qPCR. As seen in Fig. 5C to F, and consistent with our *in vivo* results, levels of these mRNAs were significantly elevated. Additionally, *ATOH-1*, a gene that promotes the differentiation of ISCs into goblet cells, was significantly decreased in infected intestinal monolayer organoids (Fig. 5G).

To further investigate the PDCoV-induced inhibition of ISC differentiation into goblet cells, we used 10 μmol/L DAPT (a Notch signaling inhibitor [34]) to rescue the loss of goblet cells in PDCoV-infected intestinal monolayer organoids. In cells treated with DAPT plus PDCoV, the number of PAS-stained goblet cells was greater than in cells only infected with PDCoV (Fig. 5H). The level of *MUC-2* was also higher in the cells treated with DAPT plus PDCoV than in the cells only infected with PDCoV (Fig. 5I).

Taken together, our results show that PDCoV infection leads to the loss of goblet cells and impedes the formation of goblet cells and the secretion of mucus through activating the Notch signaling pathway both *in vitro* and *in vivo*.

## DISCUSSION

PDCoV primarily infects intestinal epithelial cells (35). Typically, IPEC-J2 cells (an intestinal porcine epithelial cell line) are utilized as a model *in vitro* to investigate the molecular epidemiology and pathogenesis of PDCoV. Although cell lines are convenient and easy to grow, they do not simulate well the natural viral infection process of the intestinal epithelium. Sato et al. first cultured a three-dimensional structure of small intestinal stem cells and called it a small intestinal organoid (31). Researchers have expanded that technology to develop various other organoid systems (36). Li et al. developed a porcine intestinal organoid culture system in which TGEV can infect intestinal monolayer organoids and trigger antiviral immune responses (37). A previous study also showed that PEDV can infect multiple cells types in porcine intestinal enteroids, including enterocytes, goblet cells, and stem cells (38). In this investigation, we employed 2D primary porcine monolayer enteroids (derived from ISCs of the jejunum) as a PDCoV infection model; based on RT-qPCR and IFA, these enteroids supported infection by PDCoV. We also found, consistent with the study by Yin et al. (39), that PDCoV infection elicited the production of type I and type III interferons (Fig. 1 and 4). In contrast, as reported by Liu et al. for LLC-PK cells, PDCoV infection inhibited IFN-λ1 production (40). Taken together, our results show that the 2D porcine intestinal organoids we developed are a superior model for studying the interaction between host and pathogens.

Goblet cells are a specialized type of epithelial cell that participates in the formation and secretion of mucus in the intestinal mucus barrier. The intestinal mucus layer plays an essential role in protecting the intestine from mechanical, chemical, and biological attack and maintaining its homeostatic balance (41). Accumulating evidence has shown that intestinal pathogens hamper the formation and function of goblet cells (42). For example, human adenovirus species C was identified as preferentially infecting a subset of goblet cells (19), enterovirus 71 infects goblet cell in 2D monolayer enteroids and reduces the expression of MUC-1 and MUC-2 (21), and PEDV infection leads to decreased numbers of goblet cells, and as a result, the intestinal mucus barrier is compromised, allowing other enteric pathogens to take advantage of the thinner mucus layer and establish infection (43, 44). In this study, we found that PDCoV infection resulted in goblet cells loss and diminished MUC-2 expression both *in vivo* (newborn piglets) and *in vitro* (porcine intestinal organoids) (Fig. 2 and 5), indicating that PDCoV infection hinders the function of goblet cells and disrupts the intestinal mucus barrier.

For the purpose of maintaining intestinal homeostasis, the intestinal epithelium is constantly being renewed. ISCs play a key role not only in maintaining the normal renewal of intestinal epithelium but also in self-repair and differentiation of intestinal epithelium after damage by bacterial or viral infection (29, 45). The Notch signaling pathway plays a role in maintaining the intestinal progenitor cell population, as well as regulating the secretory lineage of intestinal cells, including goblet cells, Paneth cells, and enteroendocrine cells (25, 46). Activation of the Notch signaling pathway inhibits the differentiation of goblet cells (47). Notch signals are triggered when ligands on adjacent cells bind to the signal-receiving cells (48). Ligand binding to receptors initiates the cleavage of Notch by an ADAM protease at site 2. Next, γ-secretase progressively cleaves the Notch extracellular truncation within the transmembrane domain. Thereafter, the Notch intracellular domain is free to translocate to the nucleus and interacts with the DNA-binding protein CSL, which initiates the transcription of the target gene (49, 50). Previous studies have reported that activation of the Notch signaling pathway in the intestine regulates the proliferation and differentiation of intestinal mucosal epithelial cells (27), and the transcription factor hairy and enhancer of split 1 (HES-1) can act as a target gene downstream of the Notch signaling pathway to inhibit the differentiation of goblet cells (51, 52). In contrast, inhibition of HES-1 leads to decreased cell proliferation and increased secretory cells (53). Various pathogens modulate Notch signaling to evade innate cellular responses or affect the differentiation of stem cells (54). Moreover, apart from regulating the differentiation of stem cells, Notch signaling also participates in the function of immune cells. Macrophages increase Notch ligand Delta-like 1 (Dll-1) expression following influenza virus infection, thereby regulating IFN-λ levels from CD4- and CD8-positive cells (55). Furthermore, Zika virus infection elicits early activation of Notch signaling, leading to an abnormal differentiation process (56). Here, we report that PDCoV infection resulted in significantly increased levels of the Notch ligand Dll-4, Notch receptor Notch-1, and Notch effector HES-1 both *in vivo* and *in vitro* and significantly decreased levels of ATOH-1 (essential for intestinal secretory cell differentiation) (57) *in vitro* and *in vitro* (Fig. 3 and 5). Our results suggest that PDCoV infection inhibits ISC differentiation to goblet cells via activation of the Notch signaling pathway.

Epithelial cells are the first cells to respond to pathogenic infections and are the primary contact site with the pathogen-infested environment (58). There are myriad immune cells in the epithelia, which directly interact with epithelial cells to safeguard intestinal homeostasis (59). The intricate interaction between epithelial cells and immune cells can not only quickly prevent and control pathogenic infections but also effectively prevent excessive immune activation from damaging tissues (60). The coculture model for studying the interaction between epithelial cells and the immune system has been well established for disease modeling *in vitro*. Respiratory syncytial virus (RSV) infection causes dramatic epithelial remodeling; in a coculture system of airway organoids/neutrophils, cytokines secreted by RSV-infected airway organoids recruit neutrophils to the infected airway organoids (61). Immune cell-derived cytokines have also been shown to influence epithelial cell differentiation. For example, organoids cocultured with preactivated T helper 2 (TH2) cells producing interleukin 4 (IL-4) and IL-13 cytokines or preactivated TH1 cells and TH17 cells producing IFN-γ and IL-17 resulted in a significant reduction in the number of ISCs and increasing numbers of transit-amplifying cells (62). Likewise, IL-22, mainly produced by TH cells and group 3 innate lymphoid cells, triggers stem cell proliferation and subsequent growth of organoids (63). Hence, to overcome the limitation of the lack of immune cells in organoids and to better understand the impact of epithelial-immune cell interactions on the intestinal mucosal barrier during viral infection, the coculture system of organoids containing immune cells is needed for further study regarding the relationship between PDCoV infection and the intestinal mucosal barrier.

**FIG 6:**
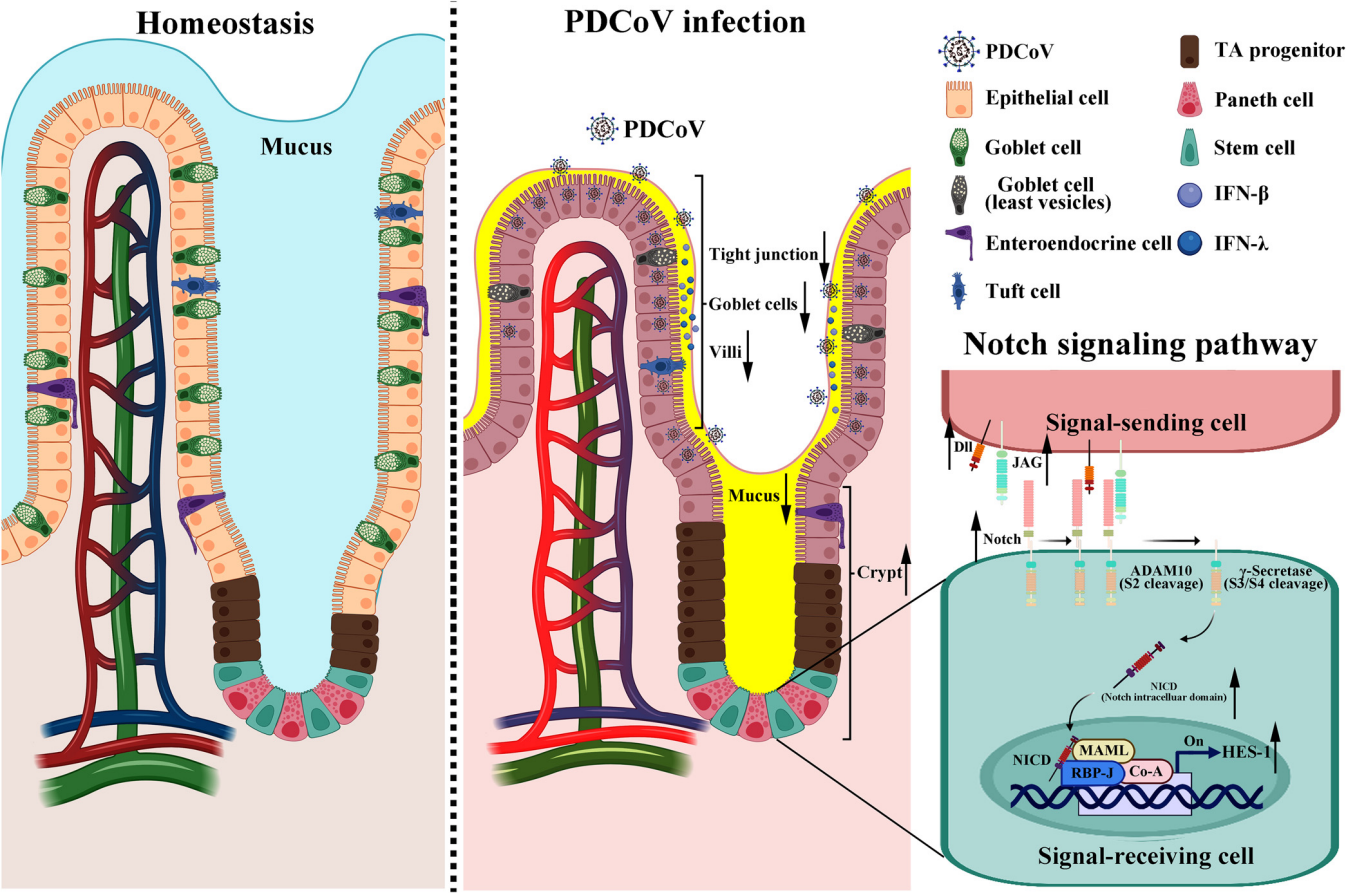
Schematic diagram of PDCoV-induced disruption of the intestinal mucosal barrier. The Notch signaling pathway is activated, goblet cell differentiation is inhibited, and mucosal secretion decreases. ↑, upregulation; ↓, downregulation.

In summary, our study highlights a strong connection between PDCoV infection and goblet cell loss. PDCoV infection severely disrupts the intestinal epithelial barrier by downregulating the mRNA expression levels of the tight-junction-related proteins (ZO-1, claudin, and occludin). More importantly, PDCoV infection results in a loss of goblet cells and decreases MUC-2 expression in both PDCoV-infected piglets and intestinal monolayer organoids. PDCoV impedes differentiation of ISCs to goblet cells by activating the Notch signaling pathway and upregulating the target gene (Fig. 6). Our findings provide a novel insight in the molecular mechanisms of PDCoV pathogenicity, and more studies are needed to explore goblet cells as novel therapeutic target cells for PDCoV infection.

## MATERIALS AND METHODS

### Virus.

PDCoV (CHN-GD16-05) was kindly provided by Zhenhai Chen from Yangzhou University.

### Animal experiments.

All animal experiments were approved by the Institutional Animal Care and Use Committee (IACUC) of the Yangzhou University Animal Experiments Ethics Committee [number SYXK (Su) IACUC 2012-0029] and followed the National Institutes of Health guidelines for the performance of animal experiments. Sunong black piglets, delivered by cesarean and deprived of colostrum, were derived from the herd maintained by the Jiangsu Academy of Agricultural Science (JAAS). The herd tested negative for antibodies to PDCoV, TGEV, PEDV, porcine reproductive and respiratory syndrome virus (PRRSV), porcine respiratory coronavirus (PRCV), and porcine circovirus type 2 (PCV2). For the PDCoV infection experiment, six newborn piglets, similar in weight, were randomly divided into two groups and housed in separate incubators; all piglets were fed 10 mL of milk every 3 h. Piglets in the PDCoV-challenged group were orogastrically inoculated with 1 × 10^6^ 50% tissue culture infective doses (TCID_50_) of PDCoV 6 h after birth. In parallel, mock-infected piglets were orogastrically administered an equal volume of phosphate-buffered saline (PBS). All pigs were observed for clinical signs of infection (vomiting, severe diarrhea, and lethargy) for 48 h and then sacrificed. Samples of small intestine were collected for Western blotting, H&E, IHC, and PAS staining.

### Western blotting.

Intestinal tissues were mechanically ground in liquid nitrogen and then suspended in radioimmunoprecipitation assay (RIPA) lysis buffer (Beyotime, China) containing protease inhibitor cocktail (Thermo Scientific, USA) at 4°C for 30 min. Samples were centrifuged at 12,000 rpm for 10 min at 4°C, and total protein concentration in the supernatants was measured by bicinchoninic acid (BCA) protein assay (Beyotime, China). A total of 20 μg of protein from each sample was resolved on a 10% SDS-PAGE gel and then transferred to a polyvinylidene difluoride (PVDF) membrane. Membranes were blocked with 5% nonfat milk in Tris-buffered saline containing 0.1% Tween 20 (TBST) and then incubated with anti-PDCoV-N (1:2,000; Medgene Labs, USA) and anti-glyceraldehyde-3-phosphate dehydrogenase (anti-GAPDH) antibodies (1:5,000; Bioworld Technology, Inc., USA) overnight at 4°C. Membranes were rinsed three times with TBST and then incubated with horseradish peroxidase (HRP)-conjugated goat anti-mouse antibody (1:5,000; Bioworld Technology, Inc.) for 1 h at room temperature. Protein bands were detected using ECL reagent (New Cell & Molecular Biotech Co., Ltd., China).

### Tissue staining.

Paraffin-embedded intestinal segments were serially sectioned into 5-μm sections. Sections were dewaxed in xylene and then rehydrated in a series of decreasing concentrations of ethanol. Sections were then stained with hematoxylin and eosin using an H&E stain kit (Solarbio, Beijing, China) according to the manufacturer’s protocol. Goblet cells were stained using a periodic acid-Schiff (PAS) stain kit (Solarbio, Beijing, China) according to the manufacturer’s instructions.

### Immunohistochemical staining.

Briefly, paraffin sections were dewaxed and rehydrated as described above. Antigen retrieval was performed by incubating sections in 10 mM citrate buffer (pH 6.0) in a decloaking chamber for 30 min at 95°C. Sections were blocked with 5% normal goat serum and then incubated with anti-PDCoV-N, MUC-2, Notch-1, or HES-1 (1:100) overnight at 4°C in a humidified chamber. For negative controls, sections were incubated in buffered saline with Tween 20 (PBST) only. An SABC-POD kit (rabbit or mouse IgG) and a peroxidase substrate kit (both from BOSTER, Wuhan, China) were used, respectively, for amplification and visualization of signal.

### RNA isolation and quantitative real-time PCR.

Intestinal tissue homogenates were added to tubes containing TRIzol and vortexed, and then total RNA was isolated using RNAiso Plus (TaKaRa, Dalian, China) according to the manufacturer’s protocol. The concentration of RNA was measured with a NanoDrop instrument (Thermo Scientific, USA). One microgram of total RNA was subjected to reverse transcription using HiScript II Q RT SuperMix (Vazyme, Nanjing, China). The amount of mRNA was determined by RT-qPCR using AceQ universal SYBR qPCR master mix (Vazyme, Nanjing, China) in an Applied Biosystems 7500 real-time PCR system (Applied Biosystems, USA). *GAPDH* served as the reference, and the relative transcript levels of target genes were calculated with the comparative threshold cycle (2^−ΔΔ^*^CT^*) method from three biological replicates. Primer-BLAST (https://www.ncbi.nlm.nih.gov/tools/primer-blast/index.cgi) was used for primer design, and primer efficiency was verified for each primer pair by melt curve analysis. Primers for specific genes are listed in Table S1 in the supplemental material.

### Transmission electron microscopy.

Jejunum segments were cut into approximately 1-mm^3^ pieces and then fixed in 2.5% glutaraldehyde for 24 h at 4°C. Tissue pieces were rinsed three times in PBS and then postfixed in 1% OsO_4_ for 1 h at room temperature. Tissue pieces were rinsed again and then dehydrated through a series of increasing ethanol and acetone concentrations. Tissues were embedded in epoxy resin and sectioned for observation by transmission electron microscopy (H-600; Hitachi, Tokyo, Japan).

### Porcine intestinal organoid culture.

Porcine jejunum crypts were isolated and cultured in a 3D organoid culture system with the support of Matrigel matrix (Corning, USA) and IntestiCult organoid growth medium (StemCell Technologies, Canada) following previously published protocols with minor modifications (31, 37). Briefly, jejuna were collected and opened longitudinally to remove luminal contents. Jejuna were cut into segments which were digested in 5 mM EDTA for 30 min on ice. After disassociation, the digestate was passed through a 70-μm filter and washed several times with cold PBS. The isolated crypt pellets were counted, and approximately 500 organoids were seeded into wells of a 24-well tissue culture plate (Corning, USA) and then incubated with Matrigel matrix in IntestiCult organoid growth medium. Growth medium was replaced every 2 to 3 days, and organoid morphology was observed daily under a microscope.

The differentiated organoids were used for development of 2D porcine intestinal monolayer organoids. Well-developed organoids were collected from Matrigel and dissociated with TrypLE Express (GIBCO, USA) for 10 min at 37°C, seeded into 48-well plates, and then incubated at 37°C in a humidified 5% CO_2_ atmosphere; the intestinal monolayer organoids reached confluence after 3 days.

### Infection of 2D intestinal monolayer organoids.

The confluent monolayer organoids were washed with PBS and incubated with PDCoV (MOI = 0.1 or 1) or Dulbecco’s modified Eagle medium (DMEM)–F-12 (mock) for 1 h at 37°C in a humidified 5% CO_2_ atmosphere and then washed three times with DMEM–F-12 to remove unattached virus. Monolayer organoids were cultured in IntestiCult organoid growth medium containing 8 μg/mL of trypsin and collected at different times postinfection for total RNA extraction or cell staining.

### Indirect immunofluorescence.

Monolayer organoids were grown on coverslips in 24-well tissue culture plates and infected with PDCoV (MOI = 1) for 24 h at 37°C, then fixed in 4% paraformaldehyde for 20 min, permeabilized with 0.1% Triton X-100 in PBS for 5 min, rinsed, and then blocked with 5% bovine serum albumin for 1 h. Organoids were incubated with anti-PDCoV-N (1:200) for 12 h at 4°C, washed three times with PBS, and then incubated with goat anti-mouse IgG H&L (1:200; Alexa Fluor 647; Beijing Biosynthesis Biotechnology Co. Ltd., China) for 30 min at room temperature. Samples were washed again three times and incubated with 1 μg/mL of 4′,6-diamidino-2-phenylindole (DAPI) for 10 min. Images were captured by a Leica TCS SP8 stimulated emission depletion (STED) laser scanning confocal microscope (Leica, Germany) and analyzed using Leica LAS AF Lite (Leica).

### TEER.

The 6-day-old spheroids were broken into single cells and then plated on 24-well transparent Transwell inserts (0.4-μm pore size; 6.5-mm diameter; Corning, USA) at a density of 2.5 × 10^4^ cells/well. The transepithelial electrical resistance (TEER) of monolayer enteroids was measured with a Millicell ERS-2 voltohmmeter (Merck Millipore, USA) as previously described (64). Resistance of a unit area was determined by the following formula: (total resistance − blank resistance) (in ohms) × effective membrane area (in square centimeters).

### Establishment of the monolayer enteroid/ST cell coculture system.

The collected monolayer enteroids were seeded at a concentration of 2.5 × 10^4^ cells/well onto the upper chambers of Transwell inserts until the TEER was >1,000 Ω ·cm^2^. The monolayer enteroids were then infected with PDCoV (MOI = 1) for 1 h at 37°C and washed three times with DMEM–F-12 to remove unattached virus. Subsequently the upper chamber assemblies were transferred to lower chambers containing confluent ST cells and cocultured for 24 h. TEER was then measured to evaluate the effect of PDCoV infection on the integrity of the monolayer enteroid barrier. Monolayer enteroids and ST cells were collected for RNA isolation.

### Statistical analysis.

All statistical analysis was performed using GraphPad Prism 6 software. Data are presented as means ± standard deviations (SD) from three independent experiments. The normality of data was tested by the Shapiro-Wilk test, and the homogeneity of variance was checked by Bartlett’s test or Brown-Forsythe test. Outliers were determined by Grubbs’ test (alpha = 0.05) and excluded from the analyses. The Student *t* test was used to compare the significant differences between PDCoV-infected and mock-infected groups. For comparisons of three or more groups, one-way analysis of variance (ANOVA) was performed. Differences were considered statistically significant at *P* values of <0.05 and highly statistically significant at *P* values of <0.01.
