# Supplementary material for: Porcine Deltacoronavirus Infection Disrupts the Intestinal Mucosal Barrier and Inhibits Intestinal Stem Cell Differentiation to Goblet Cells via the Notch Signaling Pathway
Source: J Virol. 2023 Jun 8;97(6):e00689-23. doi: 10.1128/jvi.00689-23 (PMC10308910; doi:10.1128/jvi.00689-23)
Supplement: Supplemental file 1 — Table S1. Download jvi.00689-23-s0001.docx, DOCX file, 0.02 MB [file jvi.00689-23-s0001.docx]

Table S1 Primer sequences for RT-qPCR

| Primers | Sequence (5′ - 3′) | Accession |
| --- | --- | --- |
| PDCoV-N | F: CCCAGCTCAAGGTTTCAGAG | KY363868.1 |
|  | R: ATTGGCACCAGTGCGAGACC |  |
| GAPDH | F: CACAGTCAAGGCGGAGAAC | NM_001206359.1 |
|  | R: CGTAGCACCAGCATCACC |  |
| IFN-β | F: TGCATCCTCCAAATCGCTCT | NM_001003923.1 |
|  | R: ATTGAGGAGTCCCAGGCAAC |  |
| IFN-λ | F: CCACGTCGAACTTCAGGCTT | NM_001142837.1 |
|  | R: ATGTGCAAGTCTCCACTGGT |  |
| ISG-15 | F: AGACCCACTGAGCATCCTG | NM_001128469.3 |
|  | R: CTGACACACCTGCTGCTTG |  |
| MX-1 | F: TCCAGCCACATCCCTCT | NM_214061.2 |
|  | R: GCCAGTCGTATTGGTCCTT |  |
| ZO-1 | F: AGCCCGAGGCGTGTTT | XM_021098856.1 |
|  | R: GGTGGGAGGATGCTGTTG |  |
| Occludin | F: ATCAACAAAGGCAACTCT | NM_001163647.2 |
|  | R: GCAGCAGCCATGTACTCT |  |
| Claudin | F: ACCCCAGTCAATGCCAGATA | NM_001244539.1 |
|  | R: GGCGAAGGTTTTGGATAGG |  |
| JAG-1 | F: TCCAGCAACCCCTGTCT | XM_005672699.3 |
|  | R: GAAGCACTGAGCACCGTT |  |
| NOTCH-1 | F: TGCAGAGTGAGACGGTGGA | XM_021081037.1 |
|  | R: AGAAGAGCAGCACGAAGGC |  |
| HES-1 | F: TACCCCAGCCAGTGTCAA | NM_001195231.1 |
|  | R: AATGTCCGCCTTCTCCAG |  |
| ATOH-1 | F: CACGGGCTGAACCACGCCTT | XM_003129319.4 |
|  | R: GGTACCCGCGCTTGCTTCGT |  |
| MUC-2 | F: CGGTCAAGGACGACACCATC | XM_021082584.1 |
|  | R: TGTTCCACACGAGAGCAAGG |  |
